# 1-(3,3-Dichloro­all­yloxy)-4-methyl-2-nitro­benzene

**DOI:** 10.1107/S1600536812023057

**Published:** 2012-05-31

**Authors:** Dong-mei Ren

**Affiliations:** a Security and Environment Engineering College, Capital University of Economics and Business, Beijing 10070, People’s Republic of China

## Abstract

In the title compound, C_10_H_9_Cl_2_NO_3_, the dihedral angle between the benzene ring and the plane of the nitro group is 39.1 (1)°, while that between the benzene ring and the plane through the three C and two Cl atoms of the dichloro­all­yloxy unit is 40.1 (1)°. In the crystal, C—H⋯O hydrogen bonds to the nitro groups form chains along the *b* axis. These chains are linked by inversion-related pairs of Cl⋯O inter­actions at a distance of 3.060 (3) Å, forming sheets approximately parallel to [-201] and generating *R*_2_^2^(18) rings. π–π contacts between benzene rings in adjacent sheets, with centroid–centroid distances of 3.671 (2) Å, stack mol­ecules along *c*.

## Related literature

For background to the applications of the title compound, see: Kolosov *et al.* (2002 ▶). For its synthesis, see: Walker *et al.* (2005 ▶). For bond-length data, see: Allen *et al.* (1987 ▶). For hydrogen-bond motifs, see: Bernstein *et al.* (1995 ▶).
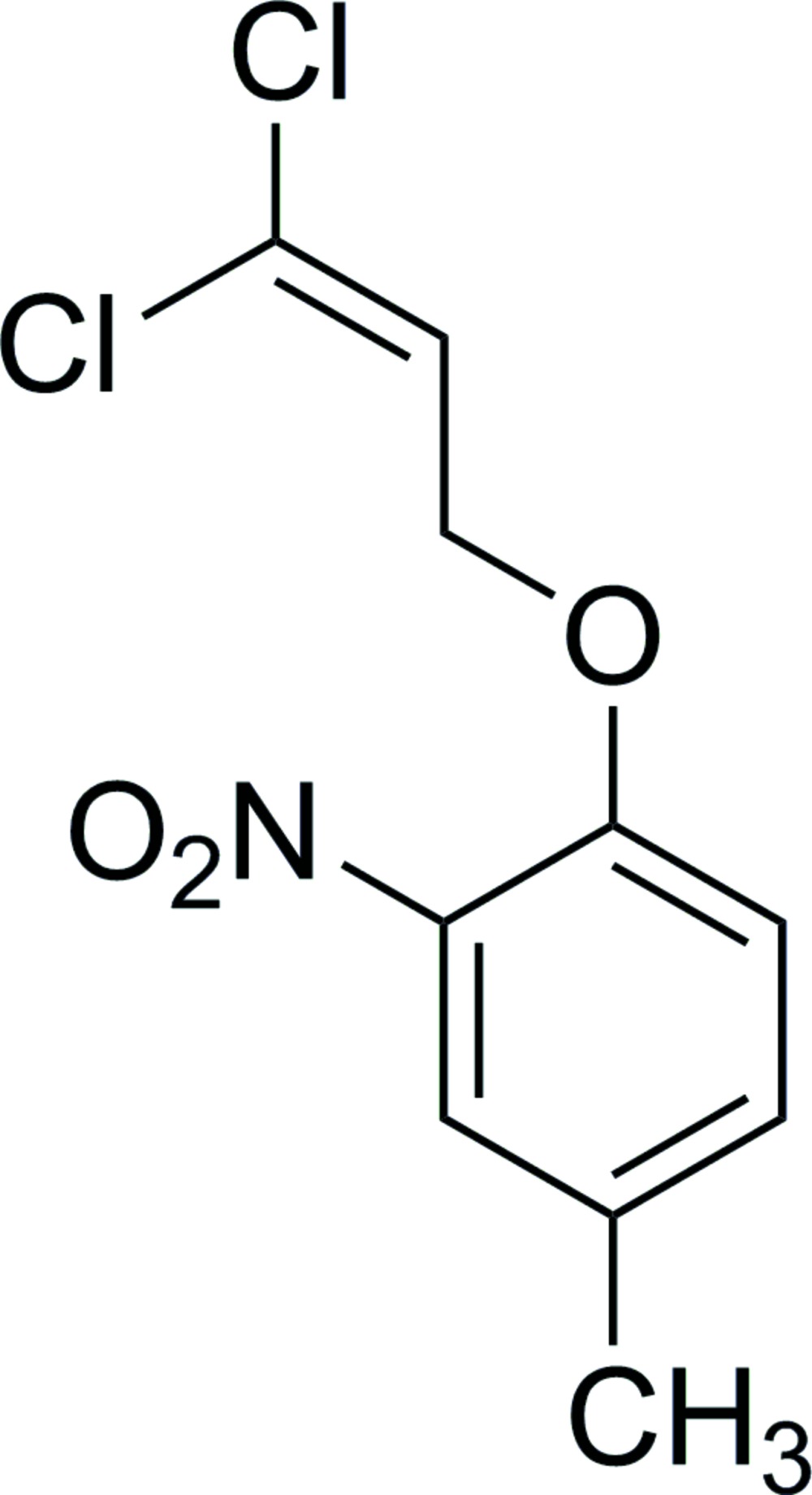


## Experimental

### 

#### Crystal data

C_10_H_9_Cl_2_NO_3_
*M* *_r_* = 262.08
Triclinic, 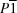

*a* = 7.5430 (15) Å
*b* = 7.7630 (16) Å
*c* = 10.713 (2) Å
α = 83.69 (3)°
β = 88.78 (3)°
γ = 67.23 (3)°
*V* = 574.8 (2) Å^3^
*Z* = 2
Mo *K*α radiation
μ = 0.56 mm^−1^
*T* = 293 K
0.30 × 0.20 × 0.10 mm

#### Data collection

Enraf–Nonius CAD-4 diffractometer
Absorption correction: ψ scan (North *et al.*, 1968 ▶) *T*_min_ = 0.851, *T*_max_ = 0.947
2277 measured reflections
2103 independent reflections
1638 reflections with *I* > 2σ(*I*)
*R*_int_ = 0.021
3 standard reflections every 200 reflections intensity decay: 1%

#### Refinement

*R*[*F* ^2^ > 2σ(*F* ^2^)] = 0.048
*wR*(*F* ^2^) = 0.156
*S* = 1.01
2103 reflections
145 parameters
H-atom parameters constrained
Δρ_max_ = 0.28 e Å^−3^
Δρ_min_ = −0.28 e Å^−3^

### 

Data collection: *CAD-4 Software* (Enraf–Nonius, 1985 ▶); cell refinement: *CAD-4 Software*; data reduction: *XCAD4* (Harms & Wocadlo, 1995 ▶); program(s) used to solve structure: *SHELXS97* (Sheldrick, 2008 ▶); program(s) used to refine structure: *SHELXL97* (Sheldrick, 2008 ▶); molecular graphics: *SHELXTL* (Sheldrick, 2008 ▶); software used to prepare material for publication: *SHELXTL*.

## Supplementary Material

Crystal structure: contains datablock(s) I, global. DOI: 10.1107/S1600536812023057/sj5235sup1.cif

Structure factors: contains datablock(s) I. DOI: 10.1107/S1600536812023057/sj5235Isup2.hkl

Supplementary material file. DOI: 10.1107/S1600536812023057/sj5235Isup3.cml

Additional supplementary materials:  crystallographic information; 3D view; checkCIF report

## Figures and Tables

**Table 1:** Hydrogen-bond geometry (Å, °)

*D*—H⋯*A*	*D*—H	H⋯*A*	*D*⋯*A*	*D*—H⋯*A*
C1—H1*A*⋯O2^i^	0.93	2.59	3.241 (4)	127

Symmetry code: (i) 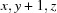.

**Fig. 1.**
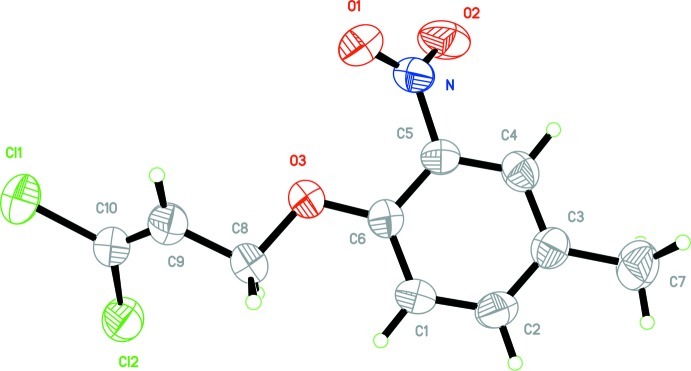
The molecular structure of (I), with the atom-numbering scheme. Displacement ellipsoids are drawn at the 50% probability level.

**Fig. 2.**
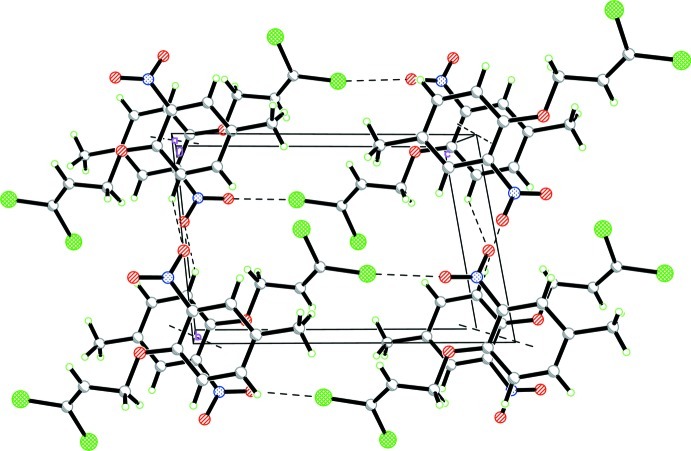
A packing diagram of (I).

## supplementary crystallographic information

### Comment

The title compound is an important intermediate in the synthesis of phenanthrenes, which can be utilized to synthesize organic semiconductors and conjugated polymers (Walker *et al.*, 2005). These materials are of wide current interest with applications in electronic and optoelectronic devices including light-emitting diodes (Kolosov *et al.*, 2002). We report here the crystal structure of the title compound, (I), as we have interests in this field.

The molecular structure of (I) is shown in Fig. 1. The dihedral angle between the (C1···C6) rings and the (C8/C9/C10/Cl1/Cl2/H9A) segment of the alloyloxy substituent is 40.1 (1)° with the nitro group (N/O1/O2) inclined at 39.1 (1)° to the ring plane. Bond distances in the molecule are normal (Allen *et al.* 1987).

In the crystal structure there is an intermolecular C1—H1A···O2 hydrogen bond that links molecules into chains along the *b* axis (Table 1, Fig. 2). Short Cl1···O1^i^ (^i^ = 1-x, -y, 1-z) contacts at a distance of 3.060 (3) Å form inversion dimers and generate R^2^_2_(18) rings (Bernstein *et al.*, 1995). These contacts link the hydrogen bonded chains into sheets approximately parallel to [-2 0 1]. Additional π···π contacts with centroid to centroid distances 3.671 (2) Å between benzene rings in adjacent sheets, stack molecules along c and generate a three dimensional network structure.

### Experimental

The title compound, (I) was prepared by a method reported in literature (Walker *et al.*, 2005). The crystals were obtained by dissolving (I) (0.1 g) in methanol (30 ml) and evaporating the solvent slowly at room temperature for about 8 d.

### Refinement

All H atoms were positioned geometrically and constrained to ride on their parent atoms, with C—H = 0.93 Å for aromatic H and 0.96 Å for alkyl H, respectively. The *U*_iso_(H) = *xU*_eq_(C), where *x* = 1.2 for aromatic H and *x* = 1.5 for other H.

### Crystal data

**Table:**

C_10_H_9_Cl_2_NO_3_	*Z* = 2
*M**_r_* = 262.08	*F*(000) = 268
Triclinic, *P*1	*D*_x_ = 1.514 Mg m^−^^3^
Hall symbol: -P 1	Mo *K*α radiation, λ = 0.71073 Å
*a* = 7.5430 (15) Å	Cell parameters from 25 reflections
*b* = 7.7630 (16) Å	θ = 10–13°
*c* = 10.713 (2) Å	µ = 0.56 mm^−^^1^
α = 83.69 (3)°	*T* = 293 K
β = 88.78 (3)°	Block, colourless
γ = 67.23 (3)°	0.30 × 0.20 × 0.10 mm
*V* = 574.8 (2) Å^3^	

### Data collection

**Table:**

Enraf–Nonius CAD-4 diffractometer	1638 reflections with *I* > 2σ(*I*)
Radiation source: fine-focus sealed tube	*R*_int_ = 0.021
Graphite monochromator	θ_max_ = 25.4°, θ_min_ = 1.9°
ω/2θ scans	*h* = 0→9
Absorption correction: ψ scan (North *et al.*, 1968)	*k* = −8→9
*T*_min_ = 0.851, *T*_max_ = 0.947	*l* = −12→12
2277 measured reflections	3 standard reflections every 200 reflections
2103 independent reflections	intensity decay: 1%

### Refinement

**Table:**

Refinement on *F*^2^	Primary atom site location: structure-invariant direct methods
Least-squares matrix: full	Secondary atom site location: difference Fourier map
*R*[*F*^2^ > 2σ(*F*^2^)] = 0.048	Hydrogen site location: inferred from neighbouring sites
*wR*(*F*^2^) = 0.156	H-atom parameters constrained
*S* = 1.01	*w* = 1/[σ^2^(*F*_o_^2^) + (0.1*P*)^2^ + 0.130*P*] where *P* = (*F*_o_^2^ + 2*F*_c_^2^)/3
2103 reflections	(Δ/σ)_max_ < 0.001
145 parameters	Δρ_max_ = 0.28 e Å^−^^3^
0 restraints	Δρ_min_ = −0.28 e Å^−^^3^

### Special details

**Table:**

Geometry. All e.s.d.'s (except the e.s.d. in the dihedral angle between two l.s. planes) are estimated using the full covariance matrix. The cell e.s.d.'s are taken into account individually in the estimation of e.s.d.'s in distances, angles and torsion angles; correlations between e.s.d.'s in cell parameters are only used when they are defined by crystal symmetry. An approximate (isotropic) treatment of cell e.s.d.'s is used for estimating e.s.d.'s involving l.s. planes.
Refinement. Refinement of *F*^2^ against ALL reflections. The weighted *R*-factor *wR* and goodness of fit *S* are based on *F*^2^, conventional *R*-factors *R* are based on *F*, with *F* set to zero for negative *F*^2^. The threshold expression of *F*^2^ > σ(*F*^2^) is used only for calculating *R*-factors(gt) *etc*. and is not relevant to the choice of reflections for refinement. *R*-factors based on *F*^2^ are statistically about twice as large as those based on *F*, and *R*- factors based on ALL data will be even larger.

### Fractional atomic coordinates and isotropic or equivalent isotropic displacement parameters (Å^2^)

**Table:**

	*x*	*y*	*z*	*U*_iso_*/*U*_eq_	
Cl1	0.35049 (14)	0.31042 (13)	0.40617 (7)	0.0723 (3)	
Cl2	0.20254 (13)	0.53282 (12)	0.60994 (8)	0.0713 (3)	
O1	0.8637 (4)	−0.2999 (3)	0.8304 (2)	0.0767 (8)	
O2	0.7766 (4)	−0.4263 (3)	0.9976 (2)	0.0731 (7)	
O3	0.6554 (3)	0.0677 (3)	0.82149 (17)	0.0542 (5)	
N	0.8110 (4)	−0.2976 (3)	0.9385 (2)	0.0534 (6)	
C1	0.6985 (4)	0.1898 (4)	1.0130 (3)	0.0506 (7)	
H1A	0.6478	0.3133	0.9755	0.061*	
C2	0.7574 (4)	0.1533 (4)	1.1372 (3)	0.0511 (7)	
H2A	0.7446	0.2537	1.1816	0.061*	
C3	0.8352 (4)	−0.0276 (4)	1.1987 (2)	0.0461 (6)	
C4	0.8481 (4)	−0.1727 (4)	1.1298 (2)	0.0457 (6)	
H4A	0.8972	−0.2958	1.1681	0.055*	
C5	0.7892 (4)	−0.1372 (4)	1.0055 (2)	0.0411 (6)	
C6	0.7139 (4)	0.0451 (4)	0.9434 (2)	0.0417 (6)	
C7	0.9018 (5)	−0.0623 (5)	1.3344 (3)	0.0661 (9)	
H7A	0.8808	0.0554	1.3652	0.099*	
H7B	1.0364	−0.1409	1.3409	0.099*	
H7C	0.8306	−0.1235	1.3835	0.099*	
C8	0.5768 (5)	0.2544 (4)	0.7592 (3)	0.0515 (7)	
H8A	0.6681	0.3142	0.7612	0.062*	
H8B	0.4597	0.3296	0.7989	0.062*	
C9	0.5358 (5)	0.2340 (4)	0.6279 (3)	0.0537 (7)	
H9A	0.6257	0.1337	0.5915	0.064*	
C10	0.3856 (5)	0.3433 (4)	0.5586 (3)	0.0517 (7)	

### Atomic displacement parameters (Å^2^)

**Table:**

	*U*^11^	*U*^22^	*U*^33^	*U*^12^	*U*^13^	*U*^23^
Cl1	0.0946 (7)	0.0785 (6)	0.0430 (4)	−0.0320 (5)	−0.0101 (4)	−0.0062 (4)
Cl2	0.0730 (6)	0.0587 (5)	0.0630 (5)	−0.0040 (4)	−0.0091 (4)	−0.0066 (4)
O1	0.112 (2)	0.0556 (14)	0.0473 (13)	−0.0128 (13)	−0.0062 (13)	−0.0175 (10)
O2	0.0858 (17)	0.0430 (12)	0.0966 (18)	−0.0307 (12)	0.0023 (14)	−0.0107 (12)
O3	0.0765 (14)	0.0393 (10)	0.0438 (11)	−0.0199 (10)	−0.0116 (10)	0.0006 (8)
N	0.0584 (15)	0.0364 (12)	0.0599 (16)	−0.0107 (11)	−0.0099 (12)	−0.0090 (11)
C1	0.0641 (18)	0.0350 (14)	0.0531 (16)	−0.0199 (13)	−0.0054 (13)	−0.0024 (12)
C2	0.0625 (18)	0.0450 (15)	0.0504 (16)	−0.0239 (13)	0.0007 (13)	−0.0127 (12)
C3	0.0460 (15)	0.0525 (16)	0.0410 (14)	−0.0199 (12)	0.0037 (11)	−0.0078 (12)
C4	0.0489 (15)	0.0438 (14)	0.0412 (14)	−0.0166 (12)	0.0006 (11)	0.0027 (11)
C5	0.0426 (14)	0.0373 (13)	0.0453 (14)	−0.0170 (11)	0.0030 (11)	−0.0065 (11)
C6	0.0453 (14)	0.0415 (14)	0.0376 (13)	−0.0165 (11)	−0.0009 (11)	−0.0020 (11)
C7	0.076 (2)	0.076 (2)	0.0444 (16)	−0.0263 (18)	−0.0025 (15)	−0.0086 (15)
C8	0.0635 (18)	0.0411 (14)	0.0446 (15)	−0.0158 (13)	−0.0059 (13)	0.0023 (12)
C9	0.0617 (18)	0.0477 (16)	0.0449 (15)	−0.0139 (14)	0.0011 (13)	−0.0051 (12)
C10	0.0648 (18)	0.0487 (16)	0.0413 (15)	−0.0218 (14)	−0.0014 (13)	−0.0033 (12)

### Geometric parameters (Å, º)

**Table:**

Cl1—C10	1.721 (3)	C3—C7	1.510 (4)
Cl2—C10	1.717 (3)	C4—C5	1.378 (4)
O1—N	1.216 (3)	C4—H4A	0.9300
O2—N	1.233 (3)	C5—C6	1.398 (4)
O3—C6	1.358 (3)	C7—H7A	0.9600
O3—C8	1.427 (3)	C7—H7B	0.9600
N—C5	1.458 (3)	C7—H7C	0.9600
C1—C2	1.375 (4)	C8—C9	1.484 (4)
C1—C6	1.383 (4)	C8—H8A	0.9700
C1—H1A	0.9300	C8—H8B	0.9700
C2—C3	1.388 (4)	C9—C10	1.307 (4)
C2—H2A	0.9300	C9—H9A	0.9300
C3—C4	1.384 (4)		
			
C6—O3—C8	117.7 (2)	O3—C6—C5	118.1 (2)
O1—N—O2	123.7 (2)	C1—C6—C5	116.9 (2)
O1—N—C5	119.6 (3)	C3—C7—H7A	109.5
O2—N—C5	116.6 (2)	C3—C7—H7B	109.5
C2—C1—C6	120.8 (3)	H7A—C7—H7B	109.5
C2—C1—H1A	119.6	C3—C7—H7C	109.5
C6—C1—H1A	119.6	H7A—C7—H7C	109.5
C1—C2—C3	122.5 (3)	H7B—C7—H7C	109.5
C1—C2—H2A	118.7	O3—C8—C9	105.5 (2)
C3—C2—H2A	118.7	O3—C8—H8A	110.6
C4—C3—C2	116.9 (2)	C9—C8—H8A	110.6
C4—C3—C7	122.2 (3)	O3—C8—H8B	110.6
C2—C3—C7	120.9 (3)	C9—C8—H8B	110.6
C5—C4—C3	120.9 (3)	H8A—C8—H8B	108.8
C5—C4—H4A	119.5	C10—C9—C8	126.3 (3)
C3—C4—H4A	119.5	C10—C9—H9A	116.9
C4—C5—C6	122.0 (3)	C8—C9—H9A	116.9
C4—C5—N	117.7 (2)	C9—C10—Cl2	123.7 (2)
C6—C5—N	120.3 (2)	C9—C10—Cl1	123.2 (2)
O3—C6—C1	124.9 (2)	Cl2—C10—Cl1	113.06 (18)
			
C6—C1—C2—C3	0.3 (5)	C8—O3—C6—C5	179.3 (2)
C1—C2—C3—C4	−1.3 (4)	C2—C1—C6—O3	178.3 (3)
C1—C2—C3—C7	178.8 (3)	C2—C1—C6—C5	0.7 (4)
C2—C3—C4—C5	1.2 (4)	C4—C5—C6—O3	−178.6 (2)
C7—C3—C4—C5	−178.9 (3)	N—C5—C6—O3	2.5 (4)
C3—C4—C5—C6	−0.2 (4)	C4—C5—C6—C1	−0.8 (4)
C3—C4—C5—N	178.7 (3)	N—C5—C6—C1	−179.7 (3)
O1—N—C5—C4	−140.1 (3)	C6—O3—C8—C9	177.3 (2)
O2—N—C5—C4	38.8 (4)	O3—C8—C9—C10	140.5 (3)
O1—N—C5—C6	38.9 (4)	C8—C9—C10—Cl2	−1.1 (5)
O2—N—C5—C6	−142.3 (3)	C8—C9—C10—Cl1	179.4 (2)
C8—O3—C6—C1	1.7 (4)		

### Hydrogen-bond geometry (Å, º)

**Table:**

*D*—H···*A*	*D*—H	H···*A*	*D*···*A*	*D*—H···*A*
C1—H1*A*···O2^i^	0.93	2.59	3.241 (4)	127

Symmetry code: (i) *x*, *y*+1, *z*.
